# SARS-CoV-2 Breakthrough Infections after introduction of 4 COVID-19 Vaccines, South Korea, 2021

**DOI:** 10.3201/eid2803.212210

**Published:** 2022-03

**Authors:** Seonju Yi, Young June Choe, Jia Kim, Yoo-Yeon Kim, Ryu Kyung Kim, Eun Jung Jang, Do Sang Lim, Hye Ryeon Byeon, Sangwon Lee, Eonjoo Park, Seung-Jin Kim, Young-Joon Park

**Affiliations:** Korea Disease Control and Prevention Agency, Cheongju, South Korea (S. Yi, J. Kim, Y.-Y. Kim, R.K. Kim, E.J. Jang, D.S .Lim, H.R. Byeon, S. Lee, E. Park, S.-J. Kim, Y.-J. Park);; Korea University Anam Hospital, Seoul, South Korea (Y.J. Choe)

**Keywords:** COVID-19, coronavirus, SARS-CoV-2, vaccine, breakthrough, coronavirus disease, severe acute respiratory syndrome coronavirus 2, respiratory infections, zoonoses, viruses, South Korea

## Abstract

We conducted a nationwide retrospective cohort study to estimate severe acute respiratory syndrome coronavirus 2 (SARS-CoV-2) breakthrough infection among recipients of 4 different vaccines in South Korea. Age-adjusted breakthrough infection rate per month was highest for Janssen (42.6/100,000 population), followed by AstraZeneca (21.7/100,000 population), Pfizer-BioNTech (8.5/100,000 population), and Moderna (1.8/100,000 population).

Since their rollout, vaccines have been highly effective globally in controlling coronavirus disease (COVID-19), caused by severe acute respiratory syndrome coronavirus 2 (SARS-CoV-2) (*1*). Breakthrough infections have been reported in some vaccine recipients, suggesting the need for public health assessment and monitoring (*2*). To date, the vaccine-specific data on breakthrough infections are limited. In early 2021, the national immunization program of South Korea introduced 4 COVID-19 vaccines: ChAdOx1 nCov-19 (AstraZeneca, https://www.astrazeneca.com), BNT162b2 (Pfizer-BioNTech, https://www.pfizer.com), Ad26.COV2.S (Johnson & Johnson/Janssen [hereafter Janssen], https://www.janssen.com), and mRNA-1273 (Moderna, https://www.moderna.com). As of October 10, 2021, a total of 70% of the country’s population have received ≥1 dose of vaccine (*3*). Introduction of the vaccines provided an opportunity to study breakthrough infections by different vaccine types. We describe a snapshot of SARS-CoV-2 breakthrough infections in South Korea and aim to identify risk by age group that might influence the observed pattern.

We conducted a nationwide retrospective cohort study to estimate SARS-CoV-2 breakthrough infection rates among AstraZeneca, Pfizer-BioNTech, Janssen, and Moderna vaccine recipients in South Korea. We included fully vaccinated persons (2 weeks past 2-dose vaccination for AstraZeneca, Pfizer-BioNTech, and Moderna vaccines; 2 weeks past 1-dose vaccination for Janssen vaccine) without history of SARS-CoV-2 infection (Appendix Figure 1). A Pfizer-BioNTech booster vaccination was offered to AstraZeneca vaccine–primed persons (2 doses of AstraZeneca vaccine, then a third dose of Pfizer-BioNTech vaccine), who were thereafter included in the analysis. Observed periods were April 7–October 10, 2021, for AstraZeneca vaccine; April 3–October 10, 2021, for Pfizer-BioNTech vaccine; June 24–October 10, 2021, for Janssen vaccine; July 30–October 10, 2021, for Moderna vaccine; and July 19–October 10, 2021, for AstraZeneca/Pfizer-BioNTech prime/booster recipients. 

We estimated breakthrough infection rate by vaccine, number of serious outcomes (cases treated with high-flow oxygen therapy, mechanical ventilator, extracorporeal membrane oxygenation, continuous renal replacement therapy, or death), and number of secondary transmissions originated from the breakthrough infection case. We identified the presence of serious outcomes through the case reporting form collected under the Infectious Disease Control and Prevention Act, which mandates epidemiologic investigation on all confirmed SARS-CoV-2 cases in South Korea. In all close contacts of laboratory-confirmed SARS-CoV-2 case-patients, we conducted epidemiologic investigations to search for the preceding link and potential onward transmission cases. We calculated age-adjusted and age-specific breakthrough infection rates as well as age-adjusted rates for serious outcomes and deaths. We randomly tested ≈20% of samples for full-length genome and spike protein sequencing to identify the presence of variant of concern.

The number of vaccinations by vaccine type are as follows: AstraZeneca (prime/booster), 8,737,343 persons; Pfizer-BioNTech (prime/booster), 10,235,891 persons; Janssen (single), 1,408,921 persons; Moderna (prime/booster), 1,190,973 persons; and AstraZeneca/Pfizer-BioNTech (prime/booster), 1,600,998 persons (Table). Age-adjusted breakthrough infection rate per month was highest among Ad26.COV2.S recipients (42.6/100,000 population), followed by AstraZeneca (prime/booster) recipients (21.7/100,000 population), AstraZeneca/Pfizer-BioNTech (prime/booster) recipients (21.3/100,000 population), Pfizer-BioNTech (prime/booster) recipients (8.5/100,000 population), and Moderna (prime/booster) recipients (1.8/100,000 population). Serious outcome (0–0.9/100,000 population) and death (0–0.2/100,000 population) after breakthrough infection were rare for all vaccine types. Secondary transmission rate was highest among Janssen recipients (19.2/100,000 population), followed by AstraZeneca (prime/booster) recipients (4.9/100,000 population). 

**Table:** Severe acute respiratory syndrome coronavirus 2 breakthrough infections by vaccine type, South Korea, 2021

Variable	ChAdOx1 nCov-19, AstraZeneca, prime/booster	BNT162b2, Pfizer-BioNTech, prime/booster	Ad26.COV2.S, Johnson & Johnson/Janssen, single dose	mRNA-1273, Moderna, prime/booster	ChAdOx1 nCov-19/BNT162b2, AstraZeneca/Pfizer-BioNTech, prime/booster
Observed period	Apr 7–Oct 10	Apr 3–Oct 10	Jun 24–Oct 1	Jul 30–Oct 10	Jul 19–Oct 10
Total vaccinations, no.	8,737,343	10,235,891	1,408,921	1,190,973	1,600,998
Breakthrough infections*	21.7	8.5	42.6	1.8	21.3
Serious outcomes*	0.3	0.2	0.9	0.0	0.1
Deaths*	0.2	0.0	0.0	0.0	0.1
Secondary transmission*	4.9	2.1	19.2	0.4	2.0

*Monthly age-adjusted rate per 100,000 population.

The highest breakthrough infection rates we observed in younger age groups were in AstraZeneca (prime/booster), Janssen (single), Moderna (prime/booster), and AstraZeneca/Pfizer-BioNTech (prime/booster) recipients (Figure). Among the Pfizer-BioNTech (prime/booster) recipients, breakthrough infection rate was highest among elderly persons 70–79 years and ≥80 years of age (Appendix Figure 2).

**Figure:**
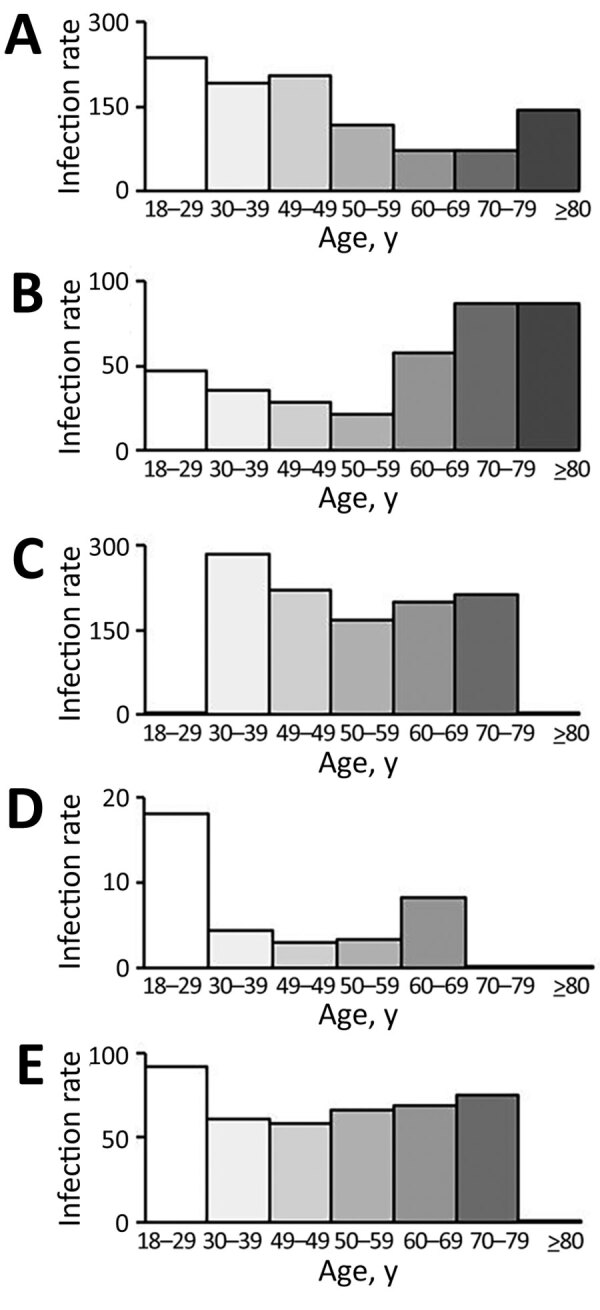
Age-specific breakthrough infection rates (cases/100,000 population) of severe acute respiratory syndrome coronavirus 2 for 4 vaccines by vaccine type, South Korea. A) ChAdOx1 nCov-19 (AstraZeneca prime/booster), April–October 2021. B) BNT162b2 (Pfizer-BioNTech prime/booster), April–October 2021. C) Ad26.COV2.S (Johnson & Johnson/Janssen, single dose), June–October 2021. D) mRNA-1273 (Moderna, prime/booster), July–October 2021. E) ChAdOx1 nCov-19/BNT162b2(AstraZeneca prime/Pfizer-BioNTech booster), July–October 2021.

We identified the variants of concern found in AstraZeneca (prime/booster) recipients as 1,285 Delta and 4 Alpha variants; in Pfizer-BioNTech (prime/booster) as 888 Delta, 14 Alpha, and 1 Beta variants; in Janssen (single), 789 Delta, 12 Alpha, and 2 Gamma variants; in Moderna (prime/booster), 13 Delta variants; and in AstraZeneca/Pfizer-BioNTech (prime/booster), 188 Delta variants.

Our findings of a higher breakthrough infection in adenovirus DNA vector vaccine recipients and lower risk among mRNA vaccine recipients are consistent with other studies. In clinical trials, 0.5% of AstraZeneca recipients (*4*) and 0.3% of Janssen recipients (*5*) had SARS-CoV-2 infections, whereas 0.05% of Pfizer-BioNTech recipients (*6*) and 0.08% of Moderna recipients (*7*) had infections. The AstraZeneca/Pfizer-BioNTech (prime/booster) recipients had breakthrough infection rate in between that of AstraZeneca (prime/booster) and Pfizer-BioNTech (prime/booster) recipients, suggesting a potential benefit from mix-and-match vaccination as observed in previous studies (*8*).

A limitation of this study is that the observed period between the vaccines were different: AstraZeneca and Pfizer-BioNTech were available for nearly 6 months, whereas Janssen and Moderna were introduced 2–3 months later. We conducted monthly adjustments of daily data; however, unidentified confounders may have affected the observed result. In addition, emergence of new variants may also affect the risk for breakthrough infection (*9*). Since mid-June 2021, Delta variant has become the dominant strain in South Korea, which may have affected vaccine effectiveness and postinfection health outcomes. Despite these limitations, our findings demonstrate uniformly low numbers of serious disease cases in recipients of all 4 vaccine types, consistent with previous findings (*10*).

In conclusion, breakthrough infection was more common among adenovirus DNA vector vaccine recipients than among mRNA vaccine recipients. Booster vaccination with mRNA vaccines in adenovirus DNA vector vaccine–primed individuals may confer additional protection against SARS-CoV-2 breakthrough infections.

Appendix Additional information about breakthrough infections of severe acute respiratory syndrome coronavirus 2, South Korea, 2021.
