# Supplementary material for: SARS-CoV-2 Breakthrough Infections after introduction of 4 COVID-19 Vaccines, South Korea, 2021
Source: Emerg Infect Dis. 2022 Mar;28(3):753–6. doi: 10.3201/eid2803.212210 (PMC8888240; doi:10.3201/eid2803.212210)
Supplement: Appendix — Additional information about breakthrough infections of severe acute respiratory syndrome coronavirus 2, South Korea, 2021. [file 21-2210-Techapp-s1.pdf]

Article DOI: https://doi.org/10.3201/eid2803.212210

SARS-CoV-2 Breakthrough Infections after Introduction of 4 COVID-19 Vaccine Types, South Korea, 2021

Appendix

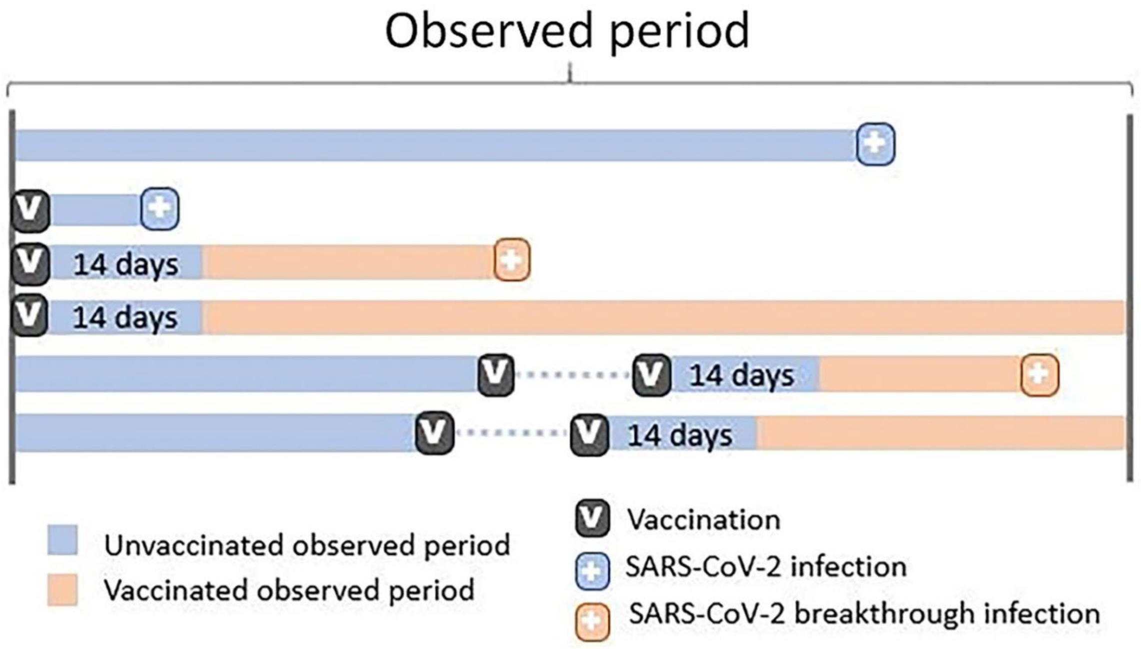

Appendix Figure 1. Timeline of selected study population for severe acute respiratory syndrome coronavirus 2 breakthrough infection, South Korea, 2021.

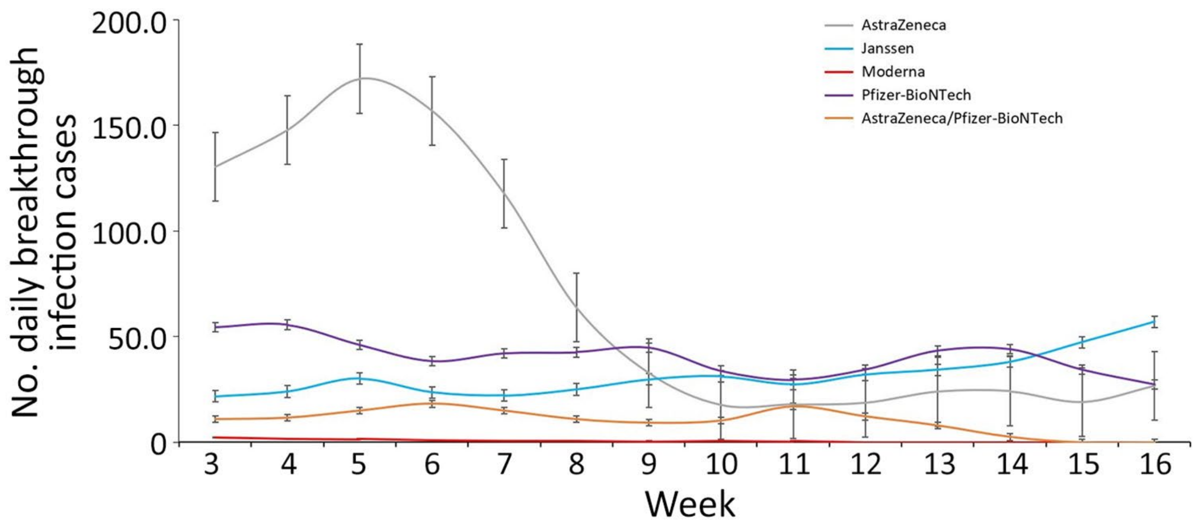

Appendix Figure 2. Daily average number of severe acute respiratory syndrome coronavirus 2 breakthrough infection cases per week, 2 weeks after full COVID-19 vaccination, South Korea, 2021.
